# Metformin Use and Leukemia Risk in Patients With Type 2 Diabetes Mellitus

**DOI:** 10.3389/fendo.2020.541090

**Published:** 2020-10-22

**Authors:** Chin-Hsiao Tseng

**Affiliations:** ^1^Department of Internal Medicine, National Taiwan University College of Medicine, Taipei, Taiwan; ^2^Division of Endocrinology and Metabolism, Department of Internal Medicine, National Taiwan University Hospital, Taipei, Taiwan; ^3^Division of Environmental Health and Occupational Medicine of the National Health Research Institutes, Zhunan, Taiwan

**Keywords:** diabetes mellitus, metformin, leukemia, National Health Insurance, Taiwan

## Abstract

**Background:**

The effect of metformin on leukemia risk remains unknown.

**Methods:**

The Taiwan’s National Health Insurance database was used to enroll 610,089 newly diagnosed type 2 diabetes patients on at least 2 anti-diabetic prescriptions during 1999–2009. We followed-up these patients until 31 December 2011, in order to determine the incidence of leukemia. We used Cox regression model (incorporated with the inverse probability of treatment-weighting using propensity scores) to estimate hazard ratios in both intention-to-treat and per-protocol analyses.

**Results:**

We enrolled 414,783 metformin initiators and 195,306 non-metformin initiators. Among them, 598 and 372 patients developed new-onset leukemia after a median follow-up period of 5.08 years and 6.79 years, respectively. The respective incidence rates were 26.52 and 28.40 per 100,000 person-years. The hazard ratio for metformin initiators versus non-metformin initiators was 0.943 (95% confidence interval 0.828–1.074) in the intention-to-treat analysis and 0.852 (95% confidence interval 0.705–1.031) in the per-protocol analysis. Sensitivity analyses after excluding patients using the exclusion criteria (a follow-up duration < 24 and < 36 months, respectively, patients with incretin-based therapies during follow-up, and patients enrolled during 2 different periods of 1999–2003 and 2004–2009) consistently showed a neutral effect. However, metformin initiators had a significantly higher risk of leukemia in the per-protocol analyses when censoring patients at a time without regular follow-up.

**Conclusion:**

Metformin use has an overall neutral effect on leukemia but we cannot exclude a significantly higher risk in patients who persistently use the drug.

## Introduction

According to a study on the global burden of cancer in 2015, the estimated number of new cases of leukemia was 606,000 and 353,000 deaths were related to leukemia, ranking it as eighth for all cancer incidences and ninth for all cancer deaths, respectively (1). Risk factors include some genetic syndromes, ionizing radiation, some environmental or occupational exposures and medications (2, 3). Diabetes patients may also suffer from a significantly higher risk of leukemia. In 2010, a Swedish study reported a significantly higher risk of leukemia in patients with type 2 diabetes mellitus (T2DM) following hospitalization (4). The estimated standardized incidence ratio compared to the general Swedish population was 1.95 (95% confidence interval: 1.72–2.17) (4). In a meta-analysis of 11 studies, the estimated odds ratio of leukemia for patients with T2DM was 1.22 (95% confidence interval: 1.03–1.44, P = 0.02) (5).

Some *in vitro* studies suggest that metformin may induce cell cycle arrest and apoptosis in leukemic cells (6, 7), and leukemic cell growth is activated by the phosphatidylinositol 3-kinase/Akt/mammalian target of rapamycin (PI3K/Akt/mTOR) pathway (8, 9). Therefore, being recognized for its activating effect on the liver kinase B1/adenosine monophosphate kinase (LKB1/AMPK) resulting in the inhibition of mTOR pathway, metformin theoretically inhibits leukemic cell growth (10, 11). However, the involvement of other pathways may also provide a protective effect of metformin on leukemia because a recent *in vitro* study suggested that metformin may suppress the growth of leukemia cells through the downregulation of AXL receptor tyrosine kinase (12).

Some investigators envisage metformin as a new adjuvant therapeutic agent for the treatment of leukemia (8, 13). However, there is lack of evidence from human studies and several clinical trials are ongoing in order to demonstrate the therapeutic effects of metformin on leukemia in patients with or without diabetes (14). On the other hand, there is paucity of data on whether metformin is preventive for the occurrence of new-onset leukemia or not. The present study investigated the effect of metformin on the risk of leukemia in T2DM patients.

## Materials and methods

The National Health Insurance (NHI) implemented since March 1995 in Taiwan covers > 99% of the population and has contracts with 93% of medical institutions and all in-hospitals nationwide. The reimbursement database of the NHI and the methods applied in the present study were described in details in previously published papers (15, 16). This database keeps all records of diseases diagnosed, medications prescribed and procedures performed. It serves as an important base for academic research after approval by an ethics review board of the National Health Research Institutes. The approval number of the present study is 99274.

The NHI database coded the different diagnoses using the International Classification of Diseases, Ninth Revision, Clinical Modification (ICD-9-CM) during the study period. Diabetes was coded 250.XX and leukemia, 204–208.

Metformin initiators [metformin (+)] and non-metformin initiators [metformin (–)] were defined according to the prescriptions of anti-diabetic drugs after diagnosis during the initial 12-month period (17). Metformin (+) were patients who had been prescribed metformin during this initial 12-month period and metformin (–) were those without any metformin prescription during this period.

**Figure 1:**
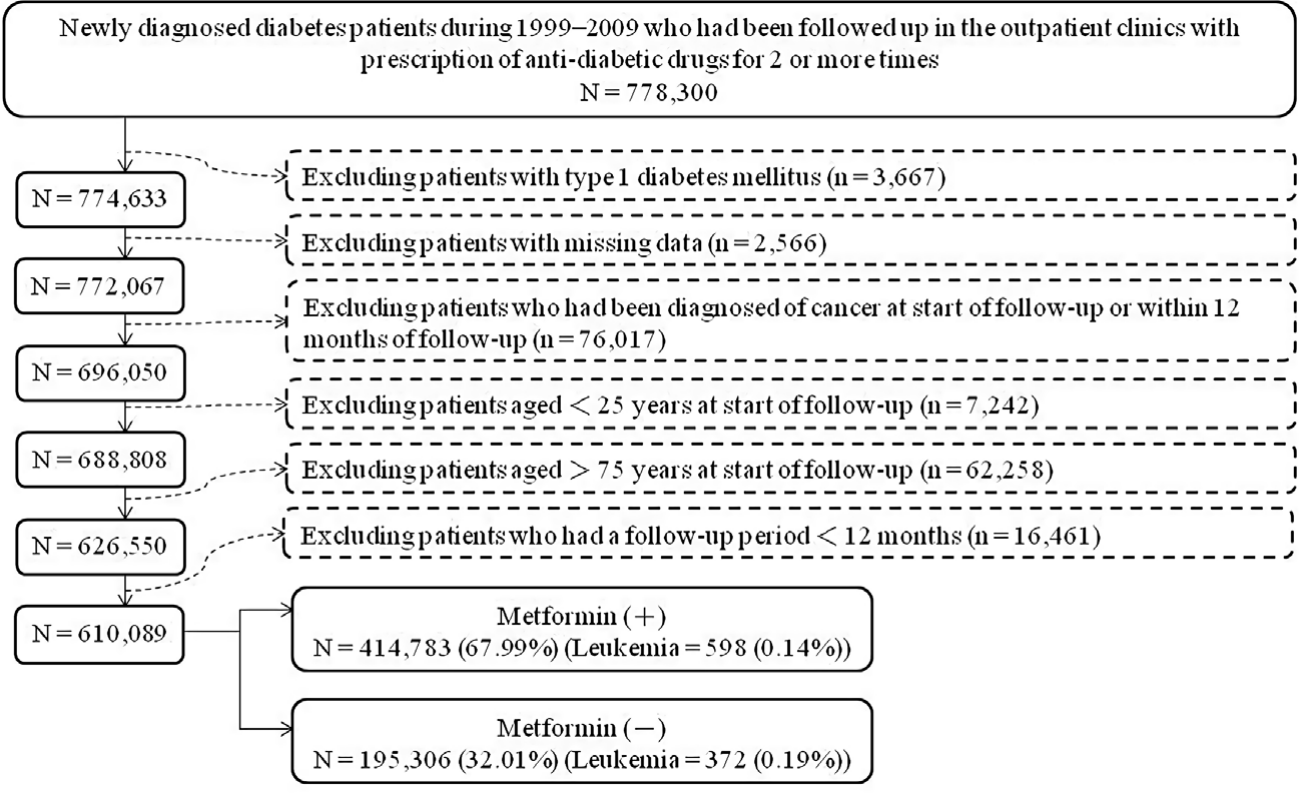
Flowchart showing the procedures followed in creating a cohort of metformin initiators [Metformin (+)] and non-metformin initiators [Metformin (–)] from the reimbursement database of Taiwan’s National Health Insurance.

**Figure 1** shows the procedures used to create metformin (+) and metformin (–). At first, we identified 778,300 newly diagnosed T2DM patients followed-up from 1999 to 2009 and on at least 2 anti-diabetic prescriptions in the outpatient clinics. The following patients were then excluded: patients with type 1 diabetes mellitus (n = 3,667), patients with missing data (n = 2,566) and patients with a diagnosis of any cancer prior to the start of follow-up or within 12 months of follow-up (n = 76,017). We also excluded patients aged < 25 years at the start of follow-up (n = 7,242), patients aged > 75 years at the start of follow-up (n = 62,258), and patients with a follow-up duration < 12 months (n = 16,461). As a result, we included 610,089 patients for analyses. Among them, 414,783 were metformin (+), and 195,306 were metformin (–).

**Table 1** shows the baseline characteristics in metformin (+) and metformin (–). These included demographic data [age, time elapsed since diabetes diagnosis (the time between diabetes diagnosis and the time of the first prescription of anti-diabetic drugs), sex, occupation and living region], major comorbidities (hypertension, dyslipidemia, and obesity), diabetes-related complications (nephropathy, eye disease, stroke, ischemic heart disease, and peripheral arterial disease), diagnoses that may be associated with cancer risk (chronic obstructive pulmonary disease, tobacco abuse, alcohol-related diagnoses, gallstone, history of Helicobacter pylori infection, Epstein-Barr virus-related diagnoses, hepatitis B virus infection, hepatitis C virus infection, diseases of the musculoskeletal system and connective tissue, and human immunodeficiency virus disease) and medications that are commonly used in diabetes patients that affect cancer risk (angiotensin converting enzyme inhibitor/angiotensin receptor blocker, calcium channel blocker, statin, fibrate, and aspirin). We classified the residence and occupation elsewhere in details (18). We coded diseases of musculoskeletal system and connective tissue as ICD-9-CM 710–739 and human immunodeficiency virus disease as 042. The ICD-9-CM codes for other diagnoses can be found in previously published papers (15, 16, 19).

**Table 1:** Baseline characteristics in non-metformin initiators and metformin initiators.

Variable	Metformin (–)		Metformin (+)		Standardized difference
	(n = 195,306)		(n = 414,783)		
	n	%	n	%	
**Demographic data**					
Age* (years)	55.61	10.92	53.85	11.07	-16.41
Time elapsed since diabetes diagnosis* (years)	1.61	1.38	1.83	1.54	17.77
Sex (men)	108,070	55.33	228,303	55.04	-0.13
Occupation**					
I	76,442	39.14	173,894	41.92	
II	42,149	21.58	94,215	22.71	3.23
III	44,014	22.54	75,968	18.32	-11.17
IV	32,701	16.74	70,706	17.05	0.77
Living region					
Taipei	60,319	30.88	143,892	34.69	
Northern	21,872	11.20	53,148	12.81	5.19
Central	34,916	17.88	74,326	17.92	0.75
Southern	34,639	17.74	62,944	15.18	-7.52
Kao-Ping and Eastern	43,560	22.30	80,473	19.40	-7.86
**Major comorbidities**					
Hypertension	115,512	59.14	250,686	60.44	3.95
Dyslipidemia	91,557	46.88	236,370	56.99	21.82
Obesity	3,837	1.96	18,177	4.38	13.28
**Diabetes-related complications**					
Nephropathy	26,286	13.46	51,212	12.35	-2.54
Eye disease	8,072	4.13	29,763	7.18	13.14
Stroke	31,085	15.92	63,101	15.21	-1.32
Ischemic heart disease	53,609	27.45	112,417	27.10	0.17
Peripheral arterial disease	19,779	10.13	45,226	10.90	3.42
**Diagnoses that may be associated with cancer risk**					
Chronic obstructive pulmonary disease	61,853	31.67	138,049	33.28	4.11
Tobacco abuse	2,112	1.08	7,487	1.81	6.19
Alcohol-related diagnoses	9,028	4.62	20,151	4.86	1.60
Gallstone	14,771	7.56	30,391	7.33	-0.89
History of Helicobacter pylori infection	28,737	14.71	65,870	15.88	3.89
Epstein-Barr virus-related diagnoses	923	0.47	2,118	0.51	0.67
Hepatitis B virus infection	1,934	0.99	6,698	1.61	5.70
Hepatitis C virus infection	4,799	2.46	10,726	2.59	1.21
Diseases of the musculoskeletal system and connective tissue	153,214	78.45	342,498	82.57	12.06
Human immunodeficiency virus disease	99	0.05	211	0.05	0.12
**Medications that are commonly used in diabetes patients or may affect cancer risk**					
Angiotensin converting enzyme inhibitor/angiotensin receptor blocker	88,148	45.13	195,569	47.15	5.03
Calcium channel blocker	87,182	44.64	173,559	41.84	-5.24
Statin	50,026	25.61	137,451	33.14	17.37
Fibrate	43,828	22.44	101,518	24.47	5.44
Aspirin	68,807	35.23	154,083	37.15	4.84

*Age and time elapsed since diabetes diagnosis are expressed as mean and standard deviation.

**Refer to Materials and Methods for the classification of occupation.

We calculated the standardized difference for each covariate as proposed by Austin and Stuart and a value > 10% was used as an indication for potential confounding in the analyses (20).

We conducted both intention-to-treat and per-protocol analyses to emulate a target trial that compares the risk of leukemia associated to the use of metformin relative to non-metformin anti-diabetic drugs. For intention-to-treat analyses, the numerator of the incidence of leukemia was the number of newly diagnosed cases during the follow-up, and the denominator was the person-years of follow-up. We commenced follow-up at the end of the initial 12-month period for the assessment of metformin (+) and metformin (–), and ended at the diagnosis of leukemia, death or the date of the last medical record by 31 December 2011 (whichever occurred first, with no exclusion according to switching to or adding other anti-diabetic drugs thereafter).

In the per-protocol analyses, we excluded patients who were not adherent to the assigned treatment within the initial 12-month period of exposure. We then followed-up the remainder for the incidence of leukemia. Follow-up started at the end of the 12-month period and ended at the first of the following events by 31 December 2011: leukemia diagnosis, death, the last reimbursement record, or non-adherence to the assigned treatment.

We used Cox regression incorporated with the inverse probability of treatment-weighting using propensity scores (PS) to estimate hazard ratios and their 95% confidence intervals for metformin (+) versus metformin (–). This method was recommended by Austin to reduce the potential confounding effect in the different distribution in characteristics (21). We created PS using the logistic regression model from the start of follow-up plus all baseline characteristics in **Table 1**. The inclusion of the start of follow-up partly accounted for some unknown risk factors: such as the introduction of novel therapeutic agents or changes in treatment guidelines during the long inclusion period.

Furthermore, we examined the consistency of findings using sensitivity analyses. Firstly, we excluded the interpretation of leukemia cases followed up for < 24 and < 36 months, respectively, in the analyses (as an effect of treatment assignment) (17). Secondly, we excluded patients treated with incretin-based therapies during follow-up to avoid their potential confounding because these therapies were introduced in Taiwan only during the follow-up period. Thirdly, we conducted analyses separately with included participants during two time intervals (1999–2003 and 2004–2009), to avoid further potential effects of some unknown risk factors such as the introduction of novel therapeutic agents and changes in treatment guidelines. Finally, we censored patients at 4 months and 6 months, respectively, after the last prescription. These would have excluded patients who had not received regular refills of anti-diabetic drugs because in Taiwan, the NHI Bureau allows a prescription of not more than 3 months each time. Because metformin use can cause anemia (22–27), associated with a higher risk of leukemia (28–30), prompting the conduction of additional sensitivity analyses after excluding patients who had ever been diagnosed of anemia (ICD-9-CM 280–285).

We performed data analyses using SAS statistical software (version 9.3, SAS Institute, Cary, NC), meanwhile we considered *P* < 0.05 as statistically significant.

## results

**Table 1** displays the baseline characteristics of metformin (–) and metformin (+). We observed standardized difference values > 10% for age, time elapsed since diabetes diagnosis, occupation, dyslipidemia, obesity, eye disease, diseases of the musculoskeletal system, and connective tissue and statin.

The median follow-up duration for metformin (–) was 6.79 years and was 5.08 years for metformin (+) in the intention-to-treat analyses. They were 2.38 and 4.58 years, respectively, in the per-protocol analyses.

**Table 2** illustrated the incidence rates of leukemia and the hazard ratios with their 95% confidence intervals comparing metformin (+) versus metformin (–) in the intention-to-treat and the per-protocol analyses. Both analyses suggested a null association between metformin use and leukemia risk. The hazard ratio in the intention-to-treat analysis was 0.943 (95% confidence interval: 0.828–1.074) and was 0.852 (95% confidence interval: 0.705–1.031) in the per-protocol analysis.

**Table 2:** Incidence rates of leukemia and hazard ratios comparing metformin initiators versus non-metformin initiators.

Model	Incident case number of leukemia	Cases followed	Person-year	Incidence rate (per 100,000 person-years)	Hazard ratio	95% Confidence interval	*P* value
**Intention-to-treat**							
Metformin (–)	372	195,306	1,309,950.45	28.40	1.000		
Metformin (+)	598	414,783	2,254,885.22	26.52	0.943	(0.828–1.074)	0.3793
**Per-protocol**							
Metformin (–)	151	195,306	620,459.93	24.34	1.000		
Metformin (+)	379	362,455	1,823,252.50	20.79	0.852	(0.705–1.031)	0.1000

Cox regression was constructed with the inverse probability of treatment-weighting using propensity scores derived from variables in **Table 1** plus the date of start of follow-up.

Sensitivity analyses in **Table 3** supported the finding of a null association in the main analyses displayed in **Table 2** (seen above). However, when censoring patients at the time of not receiving regular refills, metformin (+) had a significantly higher risk of leukemia in the per-protocol analyses (Models VI and VII). After excluding patients with a diagnosis of anemia (Model VIII), the results in the analyses did not deviate much from the main analyses shown in **Table 2**.

**Table 3:** Sensitivity analyses.

Model	Incident case number of leukemia	Cases followed	Person-year	Incidence rate (per 100,000 person-years)	Hazard ratio	95% Confidence interval	*P* value
**I. Excluding patients followed up for < 24 months**							
**Intention-to-treat**							
Metformin (–)	283	177,902	1,288,885.66	21.96	1.000		
Metformin (+)	397	346,503	2,168,791.69	18.31	0.891	(0.765–1.038)	0.1394
**Per-protocol**							
Metformin (–)	82	177,902	602,830.64	13.60	1.000		
Metformin (+)	239	310,991	1,764,390.14	13.55	0.808	(0.628–1.039)	0.0970
**II. Excluding patients followed up for < 36 months**							
**Intention-to-treat**							
Metformin (–)	230	164,514	1,255,216.89	18.32	1.000		
Metformin (+)	297	298,978	2,050,558.13	14.48	0.862	(0.725–1.024)	0.0920
**Per-protocol**							
Metformin (–)	56	164,514	578,594.22	9.68	1.000		
Metformin (+)	172	271,053	1,677,117.72	10.26	0.760	(0.562–1.029)	0.0760
**III. Excluding patients who had been treated with incretin-based therapies during follow-up**							
**Intention-to-treat**							
Metformin (–)	358	166,985	1,087,074.03	32.93	1.000		
Metformin (+)	573	343,004	1,794,891.99	31.92	0.979	(0.857–1.118)	0.7526
**Per-protocol**							
Metformin (–)	149	166,985	549,293.74	27.13	1.000		
Metformin (+)	366	292,660	1,398,347.23	26.17	0.961	(0.794–1.164)	0.6876
**IV. Patients enrolled during 1999**–**2003**							
**Intention-to-treat**							
Metformin (–)	275	110,596	920,620.00	29.87	1.000		
Metformin (+)	376	175,567	1,384,679.02	27.15	0.910	(0.779–1.063)	0.2328
**Per-protocol**							
Metformin (–)	102	110,596	392,730.35	25.97	1.000		
Metformin (+)	247	158,915	1,144,021.56	21.59	0.832	(0.657–1.053)	0.1263
**V. Patients enrolled during 2004**–**2009**							
**Intention-to-treat**							
Metformin (–)	97	84,710	389,330.46	24.91	1.000		
Metformin (+)	222	239,216	870,206.21	25.51	1.044	(0.819–1.330)	0.7299
**Per-protocol**							
Metformin (–)	49	84,710	227,729.58	21.52	1.000		
Metformin (+)	132	203,540	679,230.94	19.43	0.912	(0.656–1.269)	0.5852
**VI. Censoring patients from the time 4 months have elapsed since the last prescription**							
**Intention-to-treat**							
Metformin (–)	335	195,306	1,172,870.89	28.56	1.000		
Metformin (+)	491	414,783	1,950924.96	25.17	0.895	(0.779–1.029)	0.1203
**Per-protocol**							
Metformin (–)	135	195,306	1,172,870.89	11.51	1.000		
Metformin (+)	303	362,455	1,748,533.00	17.33	1.452	(1.185–1.780)	0.0003
**VII. Censoring patients from the time 6 months have elapsed since the last prescription**							
**Intention-to-treat**							
Metformin (–)	345	195,306	1,192,566.74	28.93	1.000		
Metformin (+)	510	414,783	1,999,911.72	25.50	0.894	(0.779–1.026)	0.1099
**Per-protocol**							
Metformin (–)	140	195,306	1,192,566.74	11.74	1.000		
Metformin (+)	319	362,455	1,796,167.77	17.76	1.456	(1.193–1.778)	0.0002
**VIII. Excluding patients with a diagnosis of anemia**							
**Intention-to-treat**							
Metformin (–)	342	191,348	1,283,062.87	26.65	1.000		
Metformin (+)	555	408,109	2,214,811.83	25.06	0.949	(0.829–1.087)	0.4499
**Per-protocol**							
Metformin (–)	137	191,348	606,481.33	22.59	1.000		
Metformin (+)	354	356,832	1,793,992.76	19.73	0.874	(0.717–1.067)	0.1865

Cox regression was constructed with the inverse probability of treatment-weighting using propensity scores derived from variables in **Table 1** plus the date of start of follow-up.

## Discussion

This is the first human study investigating the risk of leukemia after metformin use in T2DM patients. Unlike many previous studies that showed beneficial effects of metformin on the prevention of solid cancers (14) and some recent *in vitro* studies suggesting an inhibitory effect of metformin on the growth of leukemic cells (11, 12), the findings of the present study did not support a beneficial effect of metformin on leukemia in human beings (**Tables 2** and **3**). In the main analyses, the risk of leukemia was neutral while comparing metformin (+) versus metformin (–) in either the intention-to-treat or the per-protocol analysis (**Table 2**). However, from the sensitivity analyses in the per-protocol models that censored patients at the time of without regular refills, we observed a significantly increased risk among metformin (+) (Models VI and VII, **Table 3**). These sensitivity analyses (by including only patients with regular refills and adhering to metformin treatment within the desired follow-up person-years), implied a possible devastating effect of metformin on leukemia.

Deficiency in vitamin B12 (22, 23, 26, 27), folic acid (24), and/or iron (25) is a known potential long-term side effect of metformin treatment and deficiency in these micronutrients has been known to increase the risk of leukemia (28–30). Though not yet clarified, one of the possible explanations for a neutral or even devastating effect of metformin on leukemia is that the beneficial effects observed in *in vitro* studies (11, 12) could be obliterated by the deficiency in these micronutrients after long-term use of metformin.

Metformin is well known for its activation of AMPK and one of the mechanisms of preventing cancer is through its activation of AMPK resulting in the inhibition of mTOR (10, 11). A recent *in vitro* study suggested another potential mechanism through the downregulation of the AXL receptor tyrosine kinase (12). However, another recent *in vitro* and *in vivo* study suggested that only phenformin but not metformin could delay the development of T cell acute lymphoblastic leukemia/lymphoma (31). It is worth mentioning that some recent studies suggested that AMPK activators can also exert an opposite effect of promoting tumor cell survival through alternative pathways [involving redox regulation to maintain nicotinamide adenine dinucleotide phosphate (NADPH) and inhibit cell death] (32). Therefore, it is possible that metformin may exert anti-leukemic effects on one hand, but counteracted by its pro-survival pathways and its side effect of deficiency in micronutrients. The possible differentiation between solid tumors and leukemia in the activation of signaling pathways by AMPK on either the inhibition of mTOR or the activation of NADPH, could explain the different effects of metformin on the prevention of solid tumors and leukemia seen in previous observational studies. A recent study in a cancer center in New York compared the overall and disease-free survival rates in 924 diabetes patients on metformin with newly diagnosed solid tumors or acute myeloid leukemia. This analysis revealed a lack of metformin benefit in leukemia but a significant benefit for patients with solid tumors (33). The “two-faces” of AMPK on cancer has been an issue under vigorous discussion by some investigators recently (34–36). However, these speculations require further investigation for confirmation.

The present study has several clinical implications. Firstly, *in vitro* studies usually investigate a specific pathway and ignore the complex actions of and interactions with other biological pathways. Therefore, we cannot readily interpret the findings of metformin benefits on leukemic cells derived from *in vitro* studies (6, 7, 11, 12). Secondly, an overall neutral effect of metformin on leukemia risk (**Table 2**) and the potentially higher risk observed in patients in persistent use (per-protocol analyses, Models VI and VII, **Table 3**), calls for a cautious attention to the ongoing preclinical trials investigating the use of metformin as a therapeutic agent for leukemia (14). Thirdly, the possibility of differential responses to AMPK activators (such as metformin) between solid tumors and leukemia opens a new interesting venue for future research. Finally, though not proven, the hypothetical role of deficiency in vitamin B12, folic acid, or iron in the increased risk of leukemia speculated from the results (Models VI and VII, **Table 3**), calls for an attention to closely monitor levels of these micronutrients among metformin users. The timing for supplementation of these micronutrients among metformin users is an issue of clinical importance that requires vigorous research.

This study has been conducted with special attention to the potential methodological limitations commonly seen in pharmacoepidemiological studies such as selection bias, prevalent user bias, immortal time bias, and confounding by indication, as discussed previously (37).

Limitations of the study include the lack of measurement data on some potential risk factors such as hemoglobin level, serum concentrations of vitamin B12, folic acid, and iron, anthropometric factors, lifestyle, smoking, alcohol drinking, nutritional status, dietary patterns, family history, genetic markers, ionizing radiation, and environmental/occupational exposures. There are different categories of leukemia but we could not evaluate the effect of metformin on each specific category due to the lack of data. Whether the findings derived from the diabetes patients can be applied to non-diabetes ones, await additional confirmation.

In summary, this is the first observational study evaluating the effect of metformin on the risk of leukemia in humans. The findings suggest an overall neutral effect, but we cannot exclude a significantly higher risk in patients who adhered persistently to the treatment for up to five years. We recommend further studies in other populations and in non-diabetes participants in order to consolidate the findings of this study.

## Data Availability Statement

The datasets generated for this study will not be made publicly available because public availability of the dataset is restricted by local regulations to protect privacy. Requests to access the datasets should be directed to C-HT, ccktsh@ms6.hinet.net.

## Ethics Statement

The studies involving human participants were reviewed and approved by the National Health Research Institutes. Written informed consent for participation was not required for this study in accordance with the national legislation and the institutional requirements.

## Author Contributions

The author confirms being the sole contributor of this work and has approved it for publication.

## Funding

The study was partly supported by the Ministry of Science and Technology (MOST 107-2221-E-002-129-MY3) of Taiwan. The funders had no role in study design, data collection and analysis, decision to publish, or preparation of the manuscript.

## Conflict of Interest

The author declares that the research was conducted in the absence of any commercial or financial relationships that could be construed as a potential conflict of interest.
